# Symptomatic Versus Inapparent Outcome in Repeat Dengue Virus Infections Is Influenced by the Time Interval between Infections and Study Year

**DOI:** 10.1371/journal.pntd.0002357

**Published:** 2013-08-08

**Authors:** Magelda Montoya, Lionel Gresh, Juan Carlos Mercado, Katherine L. Williams, Maria José Vargas, Gamaliel Gutierrez, Guillermina Kuan, Aubree Gordon, Angel Balmaseda, Eva Harris

**Affiliations:** 1 Laboratorio Nacional de Virología, Centro Nacional de Diagnóstico y Referencia, Ministry of Health, Managua, Nicaragua; 2 Sustainable Sciences Institute, Managua, Nicaragua; 3 Division of Infectious Diseases and Vaccinology, School of Public Health, University of California, Berkeley, California, United States of America; 4 Centro de Salud Sócrates Flores Vivas, Ministry of Health, Managua, Nicaragua; 5 Division of Epidemiology, School of Public Health, University of California, Berkeley, California, United States of America; University of Rhode Island, United States of America

## Abstract

Four dengue virus serotypes (DENV1-4) circulate globally, causing more human illness than any other arthropod-borne virus. Dengue can present as a range of clinical manifestations from undifferentiated fever to Dengue Fever to severe, life-threatening syndromes. However, most DENV infections are inapparent. Yet, little is known about determinants of inapparent versus symptomatic DENV infection outcome. Here, we analyzed over 2,000 DENV infections from 2004 to 2011 in a prospective pediatric cohort study in Managua, Nicaragua. Symptomatic cases were captured at the study health center, and paired healthy annual samples were examined on a yearly basis using serological methods to identify inapparent DENV infections. Overall, inapparent and symptomatic DENV infections were equally distributed by sex. The mean age of infection was 1.2 years higher for symptomatic DENV infections as compared to inapparent infections. Although inapparent versus symptomatic outcome did not differ by infection number (first, second or third/post-second DENV infections), substantial variation in the proportion of symptomatic DENV infections among all DENV infections was observed across study years. In participants with repeat DENV infections, the time interval between a first inapparent DENV infection and a second inapparent infection was significantly shorter than the interval between a first inapparent and a second symptomatic infection. This difference was not observed in subsequent infections. This result was confirmed using two different serological techniques that measure total anti-DENV antibodies and serotype-specific neutralizing antibodies, respectively. Taken together, these findings show that, in this study, age, study year and time interval between consecutive DENV infections influence inapparent versus symptomatic infection outcome, while sex and infection number had no significant effect. Moreover, these results suggest that the window of cross-protection induced by a first infection with DENV against a second symptomatic infection is approximately 2 years. These findings are important for modeling dengue epidemics and development of vaccines.

## Author Summary

The four serotypes of the mosquito-borne dengue virus (DENV) infect an estimated 100 million humans annually, resulting in tens of millions of dengue cases and hundreds of thousands of cases of severe disease. However, infection with DENV does not always lead to clinical signs, and a large proportion of DENV infections are inapparent. Here, we studied the factors that influence whether a DENV infection is inapparent or symptomatic. Data from over 2,000 DENV infections (∼1,600 inapparent and ∼400 symptomatic) were collected during 7 years from an ongoing prospective cohort study of children in Managua, Nicaragua. We show that whether a person is infected for the first, the second, or the third time with different DENV serotypes, the proportion of symptomatic infections is similar. However, the proportion of symptomatic infection varied substantially across study years, and symptomatic infections tended to happen in older children when compared to inapparent infections. We also show that if a second DENV infection happens within a period of ∼2 years after the first infection, the second infection is more likely to be inapparent. However, if the time interval between first and second DENV infections is longer, this protection wanes and the infection is likely to be symptomatic. These findings are important for the modeling of dengue epidemics and the development of new vaccines.

## Introduction

Dengue is a major health problem globally, with more than 40% of the world's population at risk and over a hundred countries affected by epidemics [1]. In the past 50 years, the incidence of dengue has increased considerably, affecting tens of millions of people annually. Dengue is caused by an enveloped, positive-sense RNA virus in the genus *Flavivirus* of the *Flaviviridae* family, which is transmitted by mosquitoes of the *Aedes* genus. There are four serotypes of dengue virus (DENV): DENV-1, DENV-2, DENV-3 and DENV-4. Infection with DENV can be subclinical (inapparent infection) or cause a variety of clinical manifestations ranging from undifferentiated illness and Dengue Fever (DF) to severe life-threatening syndromes Dengue Hemorrhagic Fever (DHF) and Dengue Shock Syndrome (DSS) [2].

Very little is known about the determinants of inapparent versus symptomatic DENV infection outcome. By definition, inapparent infections are not detected in routine surveillance and can only be captured in the context of prospective cohort or index cluster studies. In a cohort study in Thailand, study year, total DENV infection incidence in the current and previous year, circulating DENV serotype and the number of circulating serotypes were identified as factors influencing inapparent versus symptomatic infection outcome [3], [4]. Analysis of infection outcome is further complicated by immune responses to multiple infections with different DENV serotypes, which can be either protective or pathogenic. Early experimental studies in DENV-naïve healthy volunteers showed that infection with one DENV serotype confers immunity to that particular serotype for up to 18 months [5]. In fact, this protection is thought to be life-long. On the other hand, infection with one serotype only conferred short-term (<2 months) complete protection against heterologous infection with a different serotype [5]. In Sabin's studies, heterologous protection waned over a period of several months. Heterologous protection after a short interval but not after a longer period of time was also observed in rhesus monkeys, depending on the serotype sequence [6]. In contrast, secondary heterologous infection is well documented as the single most important risk factor for severe dengue [7]–[11]. Epidemiological data from dengue epidemics in Cuba also suggest that longer time intervals between infections might increase disease severity [11]. In 1977, DENV-1 caused the first dengue epidemic in the country. This was followed by two DENV-2 epidemics caused by similar strains in 1981 and 1997, respectively [12], [13]. Interestingly, death rates were significantly higher in 1997 compared to 1981 [12]. Altogether, these observations highlight the intricate interplay between host immunity and repeat DENV infections and suggest that the time between two consecutive infections is an important factor in infection outcome.

Few studies have compared inapparent versus symptomatic outcome in primary and secondary DENV infections. In one of the first prospective dengue cohort studies in Bangkok, Thailand [9], and in a multinational index cluster study with four sites in South-East Asia and Latin America [14], the inapparent-to-symptomatic ratio was similar in primary and secondary infections. We also previously reported similar ratios in primary and secondary DENV infections in Managua, Nicaragua [15]. However, an index cluster study conducted in Kamphaeng Phet, Thailand, found very few symptomatic dengue cases among primary infections when compared to secondary infections, albeit the overall number of infections reported in the study was limited [16]. Even less is known about the impact of second, third or fourth DENV infections (collectively referred to as “secondary infections”) on inapparent versus symptomatic outcome. In fact, few reports exist in the literature of third and fourth DENV infections [17], [18]. In a hospital-based retrospective study, third and fourth DENV infections were estimated to present a lower risk of hospital admission [19]. However, once hospitalized, the risk of DHF/DSS in third and fourth DENV infections was not different from that in second DENV infections [19].

In Nicaragua, the first dengue epidemic was reported in 1985 and caused by DENV-1 and DENV-2 [20]. Several DENV-1, 2 and 4 outbreaks occurred in the early 1990's, followed by a large DENV-3 epidemic in 1994–5 [21]. Since then, all four serotypes circulate, but in contrast to hyperendemic areas, one serotype is dominant each season [22]–[24]. The dengue season starts after the first rains, with most cases occurring from August to January [15]. However, some cases are detected throughout the year. In 2004, we established the community-based, prospective Pediatric Dengue Cohort Study (PDCS) in Managua, Nicaragua [25]. Here, we analyzed serological data from all cohort participants, as well as neutralizing antibody titers in a subset of children who had experienced repeat DENV infections, using 8 annual healthy blood sample collections. We combined these results with data about dengue cases in the PDCS from 7 dengue seasons to investigate the determinants of inapparent versus symptomatic DENV infection outcome. In particular, we evaluated the impact of factors that can only be analyzed in the context of long-term cohort studies such as infection number and the time interval between infections in children with documented repeat DENV infections.

## Methods

### Ethics statement

This study was approved by the Institutional Review Boards of the Nicaraguan Ministry of Health and the University of California, Berkeley. Parents or legal guardians of all subjects provided written informed consent, and subjects 6 years of age and older provided assent.

### Study site and population

In August of 2004, a community-based pediatric dengue cohort study was established in District II of the capital city of Managua, a low-to-middle income area with a population of approximately 62,500 [25]. This ongoing study is based at the municipal Health Center Sócrates Flores Vivas (HCSFV), which is the principal source of primary health care for the district's population. Initially, participants aged 2–9 years were recruited through house-to-house visits; over time, the age range of the study was extended to 2 to 14 years of age. Each year, additional children were enrolled to maintain the age structure of the cohort [25]. Participants were encouraged to seek medical care for all illnesses through study physicians and in particular, to present early in case of a febrile episode. Cohort participants were followed closely for all illnesses, and study physicians classified participants into febrile illnesses that met the WHO dengue case definition (category A) [2], undifferentiated fever (category B), fever with an apparent focus other than dengue (category C), and non-febrile episode (category D). Children who met WHO criteria for suspected dengue (category A) as well as those with undifferentiated fever (category B) were evaluated for acute DENV infection [15], [25]. The cohort was sized such that even in years of relatively low DENV transmission, a minimum number of symptomatic cases would be identified.

### DENV infections

A suspected dengue case was considered a symptomatic DENV infection when 1) DENV RNA was detected by reverse-transcriptase polymerase chain reaction (RT-PCR) [26], [27], 2) DENV was isolated [26], 3) seroconversion was observed in paired acute and convalescent phase sera by IgM capture ELISA [26], [28], or 4) seroconversion and/or a ≥4-fold increase in total DENV-specific antibody titer in paired acute and convalescent sera was observed by Inhibition ELISA [29], [30].

Inapparent DENV infections were identified through serological testing of paired annual blood draws from healthy subjects [15], [25]. Participants whose paired annual samples demonstrated seroconversion or a 4-fold or greater increase in total DENV-specific antibody titer by Inhibition ELISA, but who had not experienced a documented febrile episode associated with acute DENV infection, were considered to have experienced an inapparent DENV infection [15], [25]. To evaluate the effectiveness of capture of febrile cases, yearly participant surveys were conducted (Table S1). Overall, surveys showed that only 1.9% of the participants reported having a fever and attending a different healthcare provider and 2.3% reported not attending any medical provider.

Both symptomatic and inapparent DENV infections were assigned a dengue season whose limits were defined by the healthy annual blood collection. As a specific date cannot be assigned to inapparent DENV infections, since by definition the infection is inapparent and thus not reported to the study health center, the inapparent infection date was assumed to be October 1^st^, during the peak of the corresponding season. For consistency, the same procedure was followed for symptomatic infections.

### Cell lines and Reporter Virus Particles

Raji-DC-SIGN cells (kind gift from B. Doranz, Integral Molecular, Philadelphia, PA) were used for all neutralization experiments. Cells were grown at 37°C at 5% CO_2_ in RPMI medium supplemented with 10% (v/v) Fetal Bovine Serum (FBS), 1% (v/v) penicillin-streptomycin, and 0.1 M HEPES (RPMI complete medium). DC-SIGN (CD209) expression was quantified by flow cytometry using a monoclonal antibody (PerCP-Cy5.5 Mouse Anti-Human CD209, BD Biosciences), and cell lots were used only if >95% of the cells were positive for DC-SIGN. DENV Reporter Viral Particles (RVP; DENV-1, Western Pacific 74; DENV-2, S16803; DENV-3, CH53489; DENV-4, TVP360) containing a GFP reporter RNA were produced by Integral Molecular as previously described [31], [32]. RVPs were stored at −80°C, and for experiments, were thawed rapidly in a water bath and kept on ice before use.

For each RVP lot, the optimal working dilution was determined. Briefly, RVPs were serially diluted 2-fold in RPMI complete medium adjusted to pH 8.0 with 5 M NaOH. Infection was carried out in a 96-well plate by mixing, in each well, 50 µl of diluted RVPs with 40,000 Raji DC-SIGN cells in a total volume of 100 µl complete RPMI media. The cells were then incubated at 37°C in 5% CO_2_ for 48 hours and subsequently fixed in 2% paraformaldehyde. The percentage of infected, GFP-expressing cells was determined by flow cytometry (Becton-Dickinson LSRII or Beckman Coulter Epics XL-MCL) using FlowJo version 7.2.5 (TreeStar Software, Ashland, OR). The highest RVP dilution yielding an infection rate of 7 to 15% was used for subsequent neutralization assays [32].

### Reporter Viral Particle-based neutralization assay

RVP neutralization assays were performed as previously described [32]. Briefly, RVPs were prepared according to the previously determined working dilution in a final volume of 25 µl of RPMI pH 8.0 complete medium. RVPs were then mixed with an equal volume of serum (eight 3-fold serial dilutions in RPMI pH 8.0 complete medium starting at 1∶5, tested in duplicate) in 96-well plates and incubated on a shaker for 1 hour at room temperature. Infections were carried out as described above. The percentage of infected, GFP-positive cells for each serum concentration was plotted as percent infection versus log_10_ of the reciprocal serum dilution using Prism 5.0 (GraphPad, La Jolla, CA). A sigmoidal dose response curve with a variable slope was then generated to determine the 50% neutralization titer, or NT_50_ – the serum dilution at which a 50% reduction in infection was observed compared to the positive (no-serum) control. Background GFP levels were subtracted from all measurements using a negative control (no-RVP).

### Neutralization assay quality control

Neutralization curves using reference sera (polyvalent anti-DENV-1+2+3+4 serum code 02/186, National Institute for Biological Standards and Control, UK) were performed with serial 2-fold dilutions of all RVP lots to ensure that viral particles were neutralized according to the law of mass action [32], [33], such that serial dilutions of RVPs yielded the same NT_50_, thus ensuring that the antibodies in the serum were in excess. Polyvalent serum was used in each neutralization assay to confirm neutralization against all 4 RVPs (neutralization control). The RVP assay was standardized both at UC Berkeley and in Nicaragua.

For each NT_50_ result, the absolute sum of squares (ABSS) and the coefficient of determination (R^2^) of the non-linear regression were calculated. If the ABSS was >0.2 and/or the R^2^ was <0.9, the data were excluded and repeated. An NT_50_ of <10 indicates a calculated NT_50_ value of <10 or the failure of the sera to neutralize at the lowest dilution by at least 50%. NT_50_ titers were independently calculated by two analysts.

### Longitudinal analysis of neutralization titers

Thirty-nine participants who entered the cohort dengue-naïve and had experienced at least two DENV infections as determined by total antibody titer measurements (ELISA) were selected. As with antibody titration by ELISA, we used annual healthy serum samples and determined the NT_50_ for all four DENV serotypes. All participants in this subset had entered the cohort between 2004 and 2007, and annual samples through 2011 were used, except for participants withdrawn from the study before then.

The following rules for interpretation of the longitudinal NT_50_ data were established and implemented. For participants who had no evidence of a previous DENV infection (i.e., NT_50_ titers for all 4 DENV serotypes in all previous years were <10), primary DENV infections were defined by seroconversion (from NT_50_<10 to NT_50_≥10) to a specific serotype. For participants with evidence of prior DENV infection, secondary DENV infections were defined by seroconversion (from NT_50_<10 to NT_50_≥40) or a ≥4-fold increase in NT_50_ (fold-change was calculated as post-infection NT_50_/pre-infection NT_50_). When several serotypes met the infection criteria during the same study year, the serotype with the highest NT_50_ fold-change was chosen. If the fold-change for more than one serotype was similar (±15%), an infection was assigned to the year but the serotype was recorded as unknown. If a symptomatic DENV infection with a given serotype was identified, no other infection with the same serotype was assigned throughout the years. If an inapparent DENV infection was identified, no other inapparent infection with the same serotype was assigned in later years. Interpretation of the DENV infection history of each participant over time was discussed by six authors to reach a consensus.

### Statistical analysis

For determination of the proportion of symptomatic DENV infections among total DENV infections, we only included symptomatic infections identified in participants who completed the study year and for whom paired annual samples were available (404 out of 448 symptomatic DENV infections). Statistical analyses were performed in STATA, version 12 (StataCorp LP, College Station, TX). The binomial test was used to assess the distribution of DENV infections by sex. Chi-square and Fisher's exact tests were used to compare categorical variables among two (or more) independent groups. The Mann-Whitney U test was used to compare intervals between consecutive DENV infections.

## Results

### Identification of DENV infections

A total of 5,541 children participated in the Pediatric Dengue Cohort Study from August 2004 to March 2011: 3,713 were enrolled at the onset of the study and 1,828 in subsequent years. We identified DENV infections during this period, corresponding to 7 dengue seasons. First, participants who met the WHO criteria for a suspected dengue case [2] and those with undifferentiated fever were evaluated for acute symptomatic DENV infection using molecular, virological, and serological diagnostic techniques (see Methods). Second, inapparent DENV infections were identified using total DENV-specific antibody titers measured by Inhibition ELISA [29], [30] in healthy annual blood samples from 8 annual collections (2004–2011). The average number of annual samples contributed per participant was 5.3±2.1 (Fig. S1A). DENV infections were stratified by study year; each year was delimited by two consecutive annual blood sample collections and encompassed a dengue season. Moreover, sequential first, second and third DENV infections were identified in participants who entered the study with no detectable anti-DENV antibodies (“naïve”). As relatively few third infections were detected, an additional category was created to study post-second DENV infections by including 1) third infections in naïve participants, and 2) second and third infections experienced by children who entered the study with anti-DENV antibodies (“non-naïve”). To identify first, second, third and post-second infections, participants who contributed two or more consecutive annual samples were included (N = 5,082). The average number of consecutive samples provided by these participants was 5.6±2.1 (Fig. S1B). The average time interval between consecutive samples was 343±41 days (Fig. S1C).

Overall, we identified 448 symptomatic and 1,606 inapparent DENV infections (Table 1). Both symptomatic and inapparent infections were equally distributed by gender. However, repeat DENV infections tended to be more frequent in males (chi-square test p = 0.060) (Table 1). We then analyzed the proportion of symptomatic DENV infections among all DENV infections. For this analysis, only participants with symptomatic DENV infections who had completed the study year were included (n = 404). The proportion of symptomatic DENV infections among all DENV infections was similar in females (20.8%) and males (19.4%, chi-square test p = 0.447). The mean age of infection was significantly higher (p<0.001), by 1.2 years, in symptomatic infections when compared to inapparent DENV infections (Table 1).

**Table 1:** Inapparent DENV infections as determined by total antibody titer and symptomatic DENV infections in cohort study, 2004–2011.

	All infections	First infection	Second infection	Third infection	Post-second infection^a^
Inapparent infections – N	1606	676	130	13	116
Female - N (%)	769 (47.9)	339 (50.2)	53 (40.8)	3 (23.8)	49 (42.2)
Age in years - mean (SD)	7.2 (2.8)	5.9 (2.5)	7.1 (2.4)	8.7 (2.1)	9.5 (2.4)
Symptomatic infections – N	448	195	37	13	58
Female - N (%)	226 (50.5)	104 (53.3)	16 (43.2)	6 (46.2)	29 (50.0)
Age in years - mean (SD)	8.4 (3.0)	7.1 (2.6)	8.2 (2.7)	9.0 (2.9)	10.4 (2.6)

a Post-second infections include third infections in dengue-naïve participants and second and third infections in dengue non-naïve participants.

### Effect of infection number and study year on inapparent versus symptomatic DENV infection outcome

**Figure 1:**
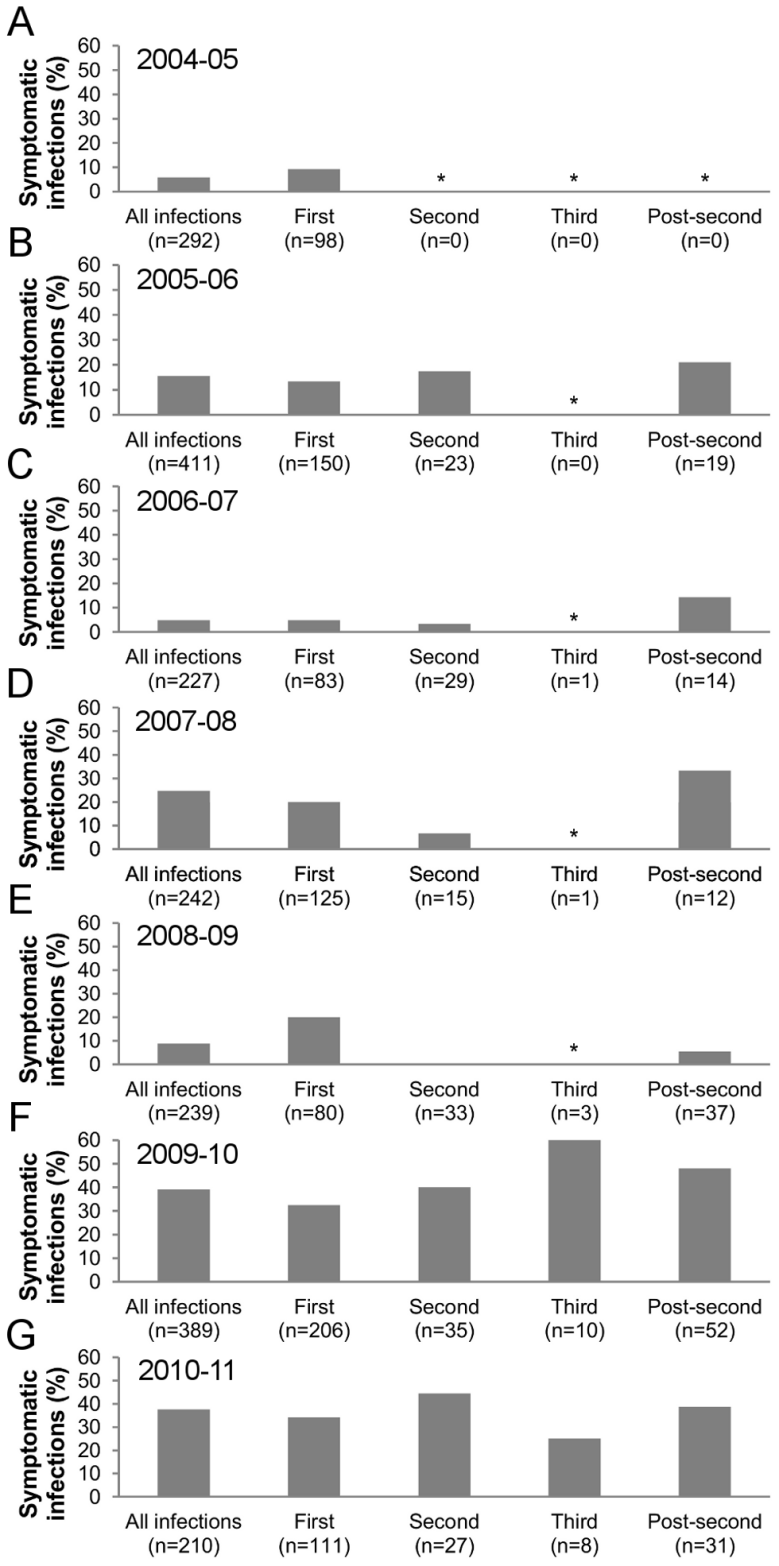
Proportion of symptomatic infections by year and infection number. (A–G) Proportion of symptomatic infections in all, first, second, third and post-second infections by study year (2004–05 to 2010–11). * The proportion of symptomatic infections was not calculated when the total number of infections per group was ≤5.

We first examined the proportion of symptomatic infections among all DENV infections per study year. This proportion showed substantial differences, ranging from 4.9% in 2006–07 to 39.1% in 2009–10 (“All infections” bars, Fig. 1A–G). Then, we analyzed the effect of infection number (first, second, third and post-second) on inapparent versus symptomatic DENV infection outcome. For each study year, trend analyses were performed with first, second and post-second DENV infections, as the number of third infections was limited. For all study years but one, the proportion of symptomatic DENV infections was similar in first, second, and post-second infections (Fisher's exact test, p>0.05, Fig. 1A–G). In 2008–09, no symptomatic second infections and very few symptomatic post-second infections were identified when compared to symptomatic first infections (Fisher's exact test, p = 0.003) (Fig. 1E). Overall, this analysis suggests that, in this study, inapparent versus symptomatic outcome is similar in first, second and post-second DENV infections.

### Interval between DENV infections according to inapparent or symptomatic outcome

For participants with repeat DENV infections, we then examined whether symptomatic versus inapparent outcome of a prior infection influences outcome of a subsequent infection. To this end, the proportion of symptomatic infections was calculated given the outcome of the previous infection. No significant difference was observed, as the proportion of symptomatic DENV infection was 24.9% when the previous infection was inapparent (N = 293) and 23.5% when the previous infection was symptomatic (N = 34) (chi-square test p = 0.859).

**Figure 2:**
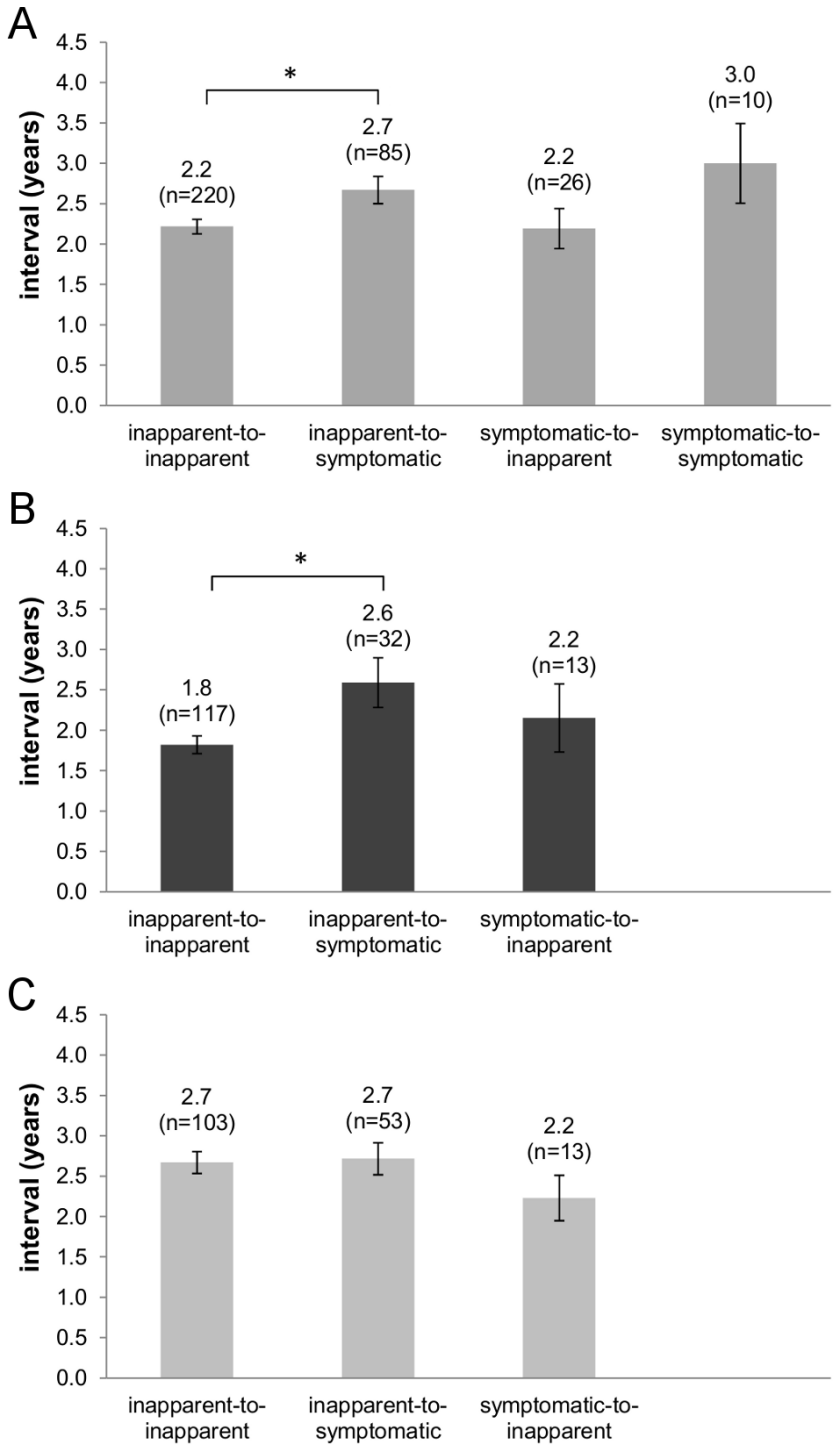
Interval between consecutive DENV infections according to inapparent or symptomatic outcome as determined by total antibody titer. The mean interval was calculated for all consecutive DENV infections (A) and stratified considering infection number into first-to-second sequences (B) and other (not first-to-second) infection (C). The inapparent-to-inapparent interval is shorter than inapparent-to-symptomatic (A) but only for first-to-second sequences (B). Error bars represent the standard error of the mean. * Mann–Whitney U test, p<0.05.

We then evaluated the effect of the time interval between infections on repeat DENV infections. The interval between two consecutive infections was defined as the number of seasons between the infections. For instance, the interval between an infection in 2005–06 and another infection in 2008–09 is 3 years. In total, 341 intervals between DENV infections were calculated. The mean interval was 2.4 years. Next, we stratified the intervals between infections with respect to the outcome of both the prior and the subsequent infection. Four different infection sequences were thus defined: an inapparent DENV infection followed by another inapparent infection (inapparent-to-inapparent) or by a symptomatic infection (inapparent-to-symptomatic), and a symptomatic DENV infection followed by an inapparent infection (symptomatic-to-inapparent) or another symptomatic infection (symptomatic-to-symptomatic). The mean interval was calculated for each of the four groups (Fig. 2A). Notably, the inapparent-to-inapparent infection mean interval was significantly shorter than the inapparent-to-symptomatic infection interval (2.2 versus 2.7 years, Mann-Whitney U test p = 0.021); all other pairwise comparisons were not significant.

We further stratified the infection sequences by infection number. Specifically, for participants who entered the cohort dengue-naïve, infection sequences were divided into “first-to-second” and “second-to-third” DENV infections. In the “first-to-second” group, the inapparent-to-inapparent infection interval was again significantly shorter than the inapparent-to-symptomatic infection interval (1.8 versus 2.6 years, Mann-Whitney U test p = 0.018) (Fig. 2B). The other pairwise comparisons were not significant. The symptomatic-to-symptomatic infection sequences were not included in the analysis as no “second-to-third” such sequence was observed. Interestingly, no difference was observed when comparing inapparent-to-inapparent and inapparent-to-symptomatic infection intervals for “second-to-third” infection sequences (2.7 versus 2.5 years, p = 0.692). Moreover, the inapparent-to-inapparent infection interval was significantly shorter in “first-to-second” (1.8 years) than in “second-to-third” infection sequences (2.7 years, Mann-Whitney U test p = 0.005). However, this observation was limited by the small number of “second-to-third” infections sequences analyzed (11 inapparent-to-inapparent and 13 inapparent-to-symptomatic).

To extend this observation, we created a new group of infection sequences by adding to the “second-to-third” sequences those infections observed in participants who entered the cohort non-dengue-naïve. This new group was termed “other infection sequences” as it includes all possible DENV infection sequences except the “first-to-second” infection group. Notably, no difference was observed between the inapparent-to-inapparent and inapparent-to-symptomatic infection intervals within this group (Fig. 2C). Furthermore, when comparing the inapparent-to-inapparent infection interval between the “first-to-second” and the “other infection sequences” groups, the former was found to be significantly shorter (1.8 versus 2.7 years, Mann-Whitney U p<0.001) (Fig. 2B–C). The symptomatic-to-symptomatic infection sequences were not included in this analysis due to the small number of observations (“first-to-second” N = 5; “other infection sequences” N = 5). Taken together, these show that the interval between two inapparent infections is significantly shorter than the inapparent-to-symptomatic infection interval, but only when considering the first and second DENV infections of a given participant.

### Longitudinal analysis of neutralizing antibody titers

We then undertook a longitudinal analysis of DENV serotype-specific neutralizing antibody titers in a subset of cohort participants. The objective of this analysis was to examine the feasibility of reconstructing participants' DENV immune history using a Reporter Viral Particle (RVP) flow cytometry-based DENV neutralization assay [32] and to substantiate the results obtained with Inhibition ELISA by measuring neutralizing antibodies instead of total anti-DENV antibodies. This assay yields reproducible serotype-specific neutralization titers that are in agreement with Plaque Reduction Neutralization Test (PRNT) results [32]. First, we examined the ability of the 50% neutralization titer (NT_50_) changes between pre- and post-infection annual samples to detect symptomatic DENV infections and to identify the correct DENV serotype in a subset of 27 confirmed symptomatic infections with serotype information available from RT-PCR and/or virus isolation. The pre- to post-infection fold-change in NT_50_ was calculated for each DENV serotype. Using the highest NT_50_ fold-change as an indicator, 26 out of 27 DENV serotypes were correctly identified (Fig. S2). In one additional case (participant M, Fig. S2), taking into account the participant's immune history allowed for the identification of the infecting serotype (DENV-3). In this case, the participant had experienced an inapparent infection with DENV-2 prior to the symptomatic episode. The NT_50_ fold-change was highest for DENV-2 but, consistent with the interpretation rules we had established, the infecting serotype was recorded as DENV-3, which had the second highest NT_50_ increase.

**Figure 3:**
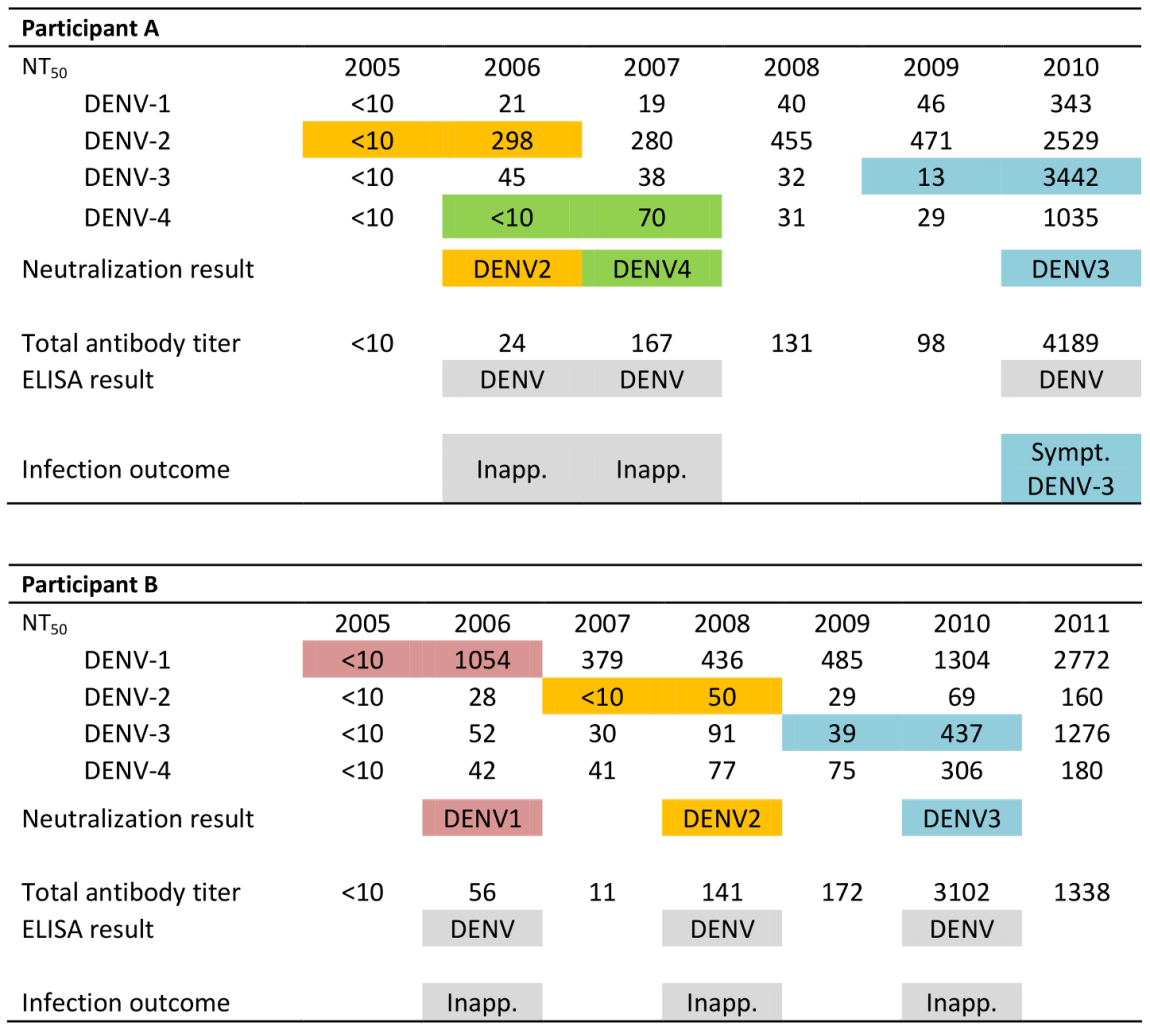
Longitudinal analysis of neutralizing antibody titers in selected cohort participants. NT_50_ for annual samples of two participants are shown as well as the interpretation of the results and the corresponding total DENV-specific antibody titer determined by Inhibition ELISA. Seroconversion or a ≥4-fold rise in antibody titer in paired annual samples was considered as indicative of a DENV infection during the study year. If the participant experienced a documented symptomatic infection, the serotype from RT-PCR/virus isolation is indicated.

Second, we analyzed longitudinal data from 39 cohort participants to determine their DENV-specific immunological history by compiling symptomatic and inapparent DENV infections as detected in consecutive annual samples (see Methods for specific rules). Longitudinal NT_50_ titers for two participants are shown in Figure 3. Both participants displayed an NT_50_<10 against all 4 serotypes in their initial sample and were therefore considered dengue-naïve. Participant A apparently experienced an inapparent DENV-2 infection in 2005–06 followed by an inapparent DENV-4 infection in 2006–07. Subsequently, NT_50_ titers did not display any major changes until 2010, when titers for all four serotypes increased more than 4-fold. However, the most likely infecting serotype was determined to be DENV-3 as the increase in NT_50_ against DENV-3 was the greatest, aside from DENV-2, which had caused the first infection. In fact, this participant experienced a symptomatic DENV-3 infection in 2009–10 as determined by RT-PCR and viral isolation using acute and convalescent samples from the period of illness. Participant B experienced 3 inapparent DENV infections: DENV-1 in 2005–06, DENV-2 in 2007–08 and DENV-3 in 2009–10. Overall, 75 inapparent DENV infections were detected among the 39 participants analyzed (Table S2). For most infections (73/75), the likely infecting serotype was identified. For the remaining two, a comparable fold-change in NT_50_ titers was observed for two serotypes, making it difficult to assign an infecting serotype.

Finally, we compared DENV serotype circulation in each study year as determined by neutralization assay using annual samples to symptomatic DENV infections detected in the entire cohort by RT-PCR and/or virus isolation. Serotype circulation was similar using both approaches, showing that the circulating serotype(s) cause both inapparent and symptomatic DENV infections and further validating the neutralization method (Fig. S3). The only striking difference was DENV-4 circulation in 2006–07, 2007–08 and 2009–10, which only caused inapparent infections. These data are consistent with limited PRNT data that we obtained as part of a study of DENV neutralizing antibodies in a random 10% of the cohort from 2004 to 2007 and in a subset of inapparent infections in different individuals each year from 2004 to 2008, where inapparent DENV-4 infections were also identified in 2006–07 and 2007–08 (M.J. Vargas, A. Balmaseda, E. Harris, unpublished results).

### Interval between DENV infections according to inapparent or symptomatic outcome as determined by neutralizing antibody titer

Using the same approach as for total antibody titers above, the intervals between consecutive DENV infections were determined in the subset of cohort participants examined using the neutralization assay. The mean interval between two DENV infections was 2.41 years (N = 54). Despite the fact that the neutralization titer dataset contained approximately 6 times fewer consecutive infection sequences than the ELISA dataset from the entire cohort, the value obtained in the neutralization subset was similar to the mean interval determined using total antibody titer (2.35 years).

**Figure 4:**
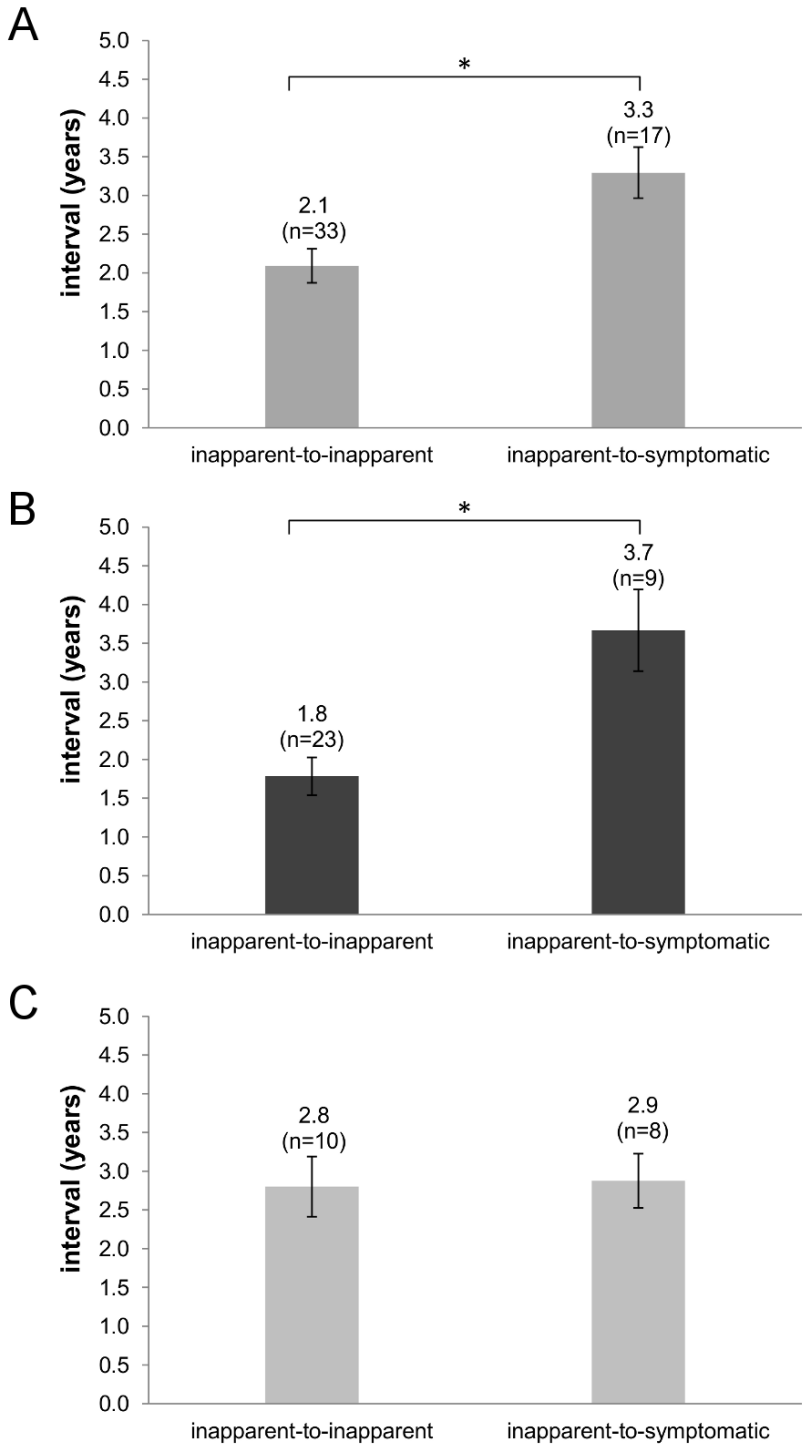
Interval between DENV infections according to inapparent or symptomatic outcome as determined by neutralizing antibody titer. The mean interval was calculated for all consecutive infections (A) and stratified considering infection number into first-to-second (B) and second-to-third (C) sequences. The inapparent-to-inapparent interval is shorter than inapparent-to-symptomatic (A) but only for first-to-second sequences (B). Error bars represent the standard error of the mean. * Mann–Whitney U test, p<0.05.

We then stratified the infection sequences by infection outcome and infection number. Only inapparent-to-inapparent and inapparent-to-symptomatic infection sequences were compared, as the number of symptomatic-to-inapparent infections was small (N = 4) and no symptomatic-to-symptomatic infection sequences were observed. When comparing all intervals, the inapparent-to-inapparent infection interval was significantly shorter than the inapparent-to-symptomatic infection interval (Mann-Whitney U test p = 0.025) (Fig. 4A). However, when we stratified by infection number, this difference was only observed in “first-to-second” subset (Mann-Whitney U test p = 0.003, Fig. 4B) and not when considering “second-to-third” infection sequences (Fig. 4C). These results corroborate the findings obtained with consecutive DENV infection interval using total antibody titers in the entire cohort.

## Discussion

In this study, we analyzed several determinants of inapparent versus symptomatic DENV infection, taking advantage of our long-term Pediatric Dengue Cohort Study in Managua, Nicaragua. Data from 1,606 inapparent and 448 symptomatic DENV infections were collected over 7 years using annual total anti-DENV antibody titers as measured by Inhibition ELISA and “enhanced” passive surveillance of febrile cases, respectively. Overall, symptomatic DENV infections were equally distributed by gender but more frequent in older children. The proportion of symptomatic DENV infections among all DENV infections varied substantially across study years but was not significantly affected by infection number (i.e., first, second, third, or post-second infections). In participants with documented repeat DENV infections, the outcome of a previous DENV infection did not influence the outcome of the subsequent infection; however, the time interval between two consecutive infections did. In fact, the interval between two inapparent DENV infections was significantly shorter that the interval between an inapparent and a symptomatic infection. However, this result was only observed when considering the first and second DENV infections of a given participant. Moreover, this finding was confirmed using a flow cytometry-based neutralization assay to quantify serotype-specific anti-DENV neutralizing antibodies in a subset of cohort participants.

The proportion of symptomatic DENV infections among total infections was found to be similar in females and males, consistent with observations in other studies [3], [14]. However, age played a role in influencing symptomatic outcome, as symptomatic DENV infections tended to occur more frequently in older children. Interestingly, this effect was not observed in the Kamphaeng Phet (Thailand) cohort [3]. The most striking determinant of infection outcome was the study year. We had previously reported large variations in the proportion of symptomatic DENV infections in the first four dengue seasons of the Pediatric Dengue Cohort Study (2004–05 to 2007–08) [15]. Here, we extended this analysis through 2010–11 and found even more dramatic variations, from ∼5–6% in 2004–05 and 2006–07 to almost 40% in 2009–10 and 2010–11. Similar temporal variations have been reported in other studies in Peru [34] and Thailand [3], [4], [35]. The factor(s) driving these differences in our Nicaraguan cohort are not completely known, although in 2007–08 a clade replacement within DENV-2 is thought to have contributed to the higher proportion of symptomatic infections [24], and in 2009–10 the concurrent H1N1 influenza pandemic may have played a role [23]. Overall, we did not observe a correlation between circulating serotypes and infection outcome, except for DENV-4, which caused mostly inapparent infections. In the cohort study from 2004 to 2011, only one DENV-4 symptomatic infection was reported. However, in the subset of 39 participants who were analyzed using the serotype-specific neutralization assay, 9 inapparent DENV-4 infections were detected, suggesting that DENV-4 does circulate in Managua but rarely causes symptomatic infections.

Conventionally, DENV infections have been defined as primary or secondary depending on the immune response profile in acute and convalescent samples [2]. No distinction is usually made between second, third and fourth DENV infections, as differences in the immune response between these categories are notoriously difficult to determine. Studying specifically first versus second versus third versus fourth DENV infections requires long-term cohort studies that capture both inapparent and symptomatic infections in the same individuals over time. Here, we report inapparent versus symptomatic outcome in first, second and third DENV infections. As the number of third infections was relatively small, we also analyzed outcome in post-second infections. Interestingly, when stratified by study year, the proportion of symptomatic DENV infections was similar in first, second, third, and post-second infections. The data provided here about post-second and third infections are important, as models suggest that post-second infections could impact dengue dynamics, overall force of infection, and incidence rates of severe dengue disease [36]. However, to date, few models have been able to incorporate information about infection number for lack of specific data about second versus third versus fourth DENV infections. In addition, there are implications for vaccine development. If, in fact, there is substantial symptomatic disease in post-second infections, then tetravalent or at least trivalent seroconversion after vaccination would be crucial for effective vaccine protection.

Both seminal observations by Sabin [5] and epidemiological reports [12], [13], [37] suggest that the time interval between consecutive DENV infections plays a role in infection outcome and severity. Here, we analyzed the time interval between repeat DENV infections and evaluated its impact on inapparent versus symptomatic outcome. As healthy blood samples were collected annually in this study, the intervals between consecutive DENV infections were calculated as integers representing annual increments. The mean interval between two DENV infections in our entire dataset was 2.4 years. We found that after an inapparent DENV infection, the interval to a subsequent inapparent DENV infection was significantly shorter than the interval to a subsequent symptomatic DENV infection (2.2 versus 2.7 years, p = 0.021). Similar numbers were obtained when the preceding infection was symptomatic, although the number of observations was small and the difference was not significant. Interestingly, the shorter inapparent-to-symptomatic infection interval was only observed when, for a given participant, the preceding infection was his/her first DENV infection and the subsequent infection the second. In this case, the inapparent-to-inapparent interval was 1.8 years versus 2.6 years for inapparent-to-symptomatic infection. These results suggest that the immunity induced by a first infection with DENV protects against a second symptomatic infection for approximately 2 years. Then, immunity wanes and is no longer protective. However, we cannot exclude that confounding factors such as age and yearly serotype-specific infection rates may contribute to the observed differences between inapparent-to-inapparent and inapparent-to-symptomatic intervals. These results are consistent with the time interval of cross-protection estimated between DENV-1 and DENV-2 infections in Nicaragua in 2005–08 [23]. These findings are also consistent with Sabin's observations, although the protection window of a few months described in his experimental study is shorter [5]. To the best of our knowledge, this is the first published report measuring the specific time interval of cross-protection prior to a subsequent DENV infection in the context of natural DENV infections.

It is well-established that secondary heterotypic DENV infection is the most important risk factor for severe disease [7]–[11]. In our cohort study, a similar effect is observed: 3.0% of secondary DENV infections were classified as DHF/DSS as compared to only 0.8% of primary infections. However, the total number of DHF/DSS cases identified in the study (n = 42) was too small to stratify them by first versus second versus third (or post-second) infections and to evaluate the impact of the time interval between consecutive DENV infections on disease severity.

The dengue plaque reduction neutralization test (PRNT) is currently considered the “gold standard” to quantify serotype-specific anti-DENV neutralizing antibodies, although it has not been well-standardized across difference laboratories in terms of reagents and testing conditions [38]–[40]. However, the size and longevity of the Pediatric Dengue Cohort Study make it logistically unfeasible to use PRNT for annual serological testing. Here, we used two serological techniques. First, to measure total anti-DENV antibodies in the large number of annual samples collected, we used the Inhibition ELISA [29], [30]. The Inhibition ELISA has been previously evaluated in Nicaragua and showed a sensitivity of 98.9% and a specificity of 100% as compared to the Hemagglutinin Inhibition assay [29]. Although the Inhibition ELISA is a fast and reliable technique, it does not provide serotype information nor does it specifically measure neutralizing anti-DENV antibodies. Thus, we used a second serological assay: the Reporter Viral Particle (RVP) flow cytometry-based DENV neutralization assay in a subset of participants. This technique has been previously evaluated and generate neutralization titers that are in good statistical agreement with PRNT [32]. A thorough quality control procedure was implemented at all steps of the assay from reagent control to data analysis. Specific rules were established to infer DENV infections from the annual sample neutralization titers. Using this set of rules, all symptomatic DENV infections identified in the subset of cohort participants were correctly captured using the RVP-generated neutralization titers. Furthermore, comparison of the serotype identified by RT-PCR and/or virus isolation and the serotype identified using NT_50_ values was 100% concordant. However, the throughput of the flow cytometry-based neutralization technique is limited compared to Inhibition ELISA, and we were only able to use it to analyze a subset of samples. The neutralization antibody data was used to confirm our findings on the time interval between repeat DENV infections. Notably, the intervals calculated using the neutralization assay closely matched those obtained using Inhibition ELISA data.

One of the limitations of this study is that serotype information is available for only a subset of the inapparent DENV infections – those processed using the RVP-based neutralization assay. We are currently expanding the number of annual samples processed using this technique. This will allow us to address several unanswered questions regarding inapparent versus symptomatic DENV infection outcome, including the impact of DENV serotype and the sequence of DENV serotypes on outcome and the effect of the magnitude and breadth of pre-infection neutralizing titers on infection outcome. Another limitation is the particular epidemiological context of dengue epidemics in Nicaragua. In contrast to hyperendemic areas in Asia where all four DENV serotypes circulate simultaneously, in Nicaragua one serotype predominates in each dengue season [22]–[24]. Moreover, a substantial amount of symptomatic infections reported in this study occurred in 2009–10 and 2010–11, when DENV-3 was the main circulating serotype, and this could conceivably influence the determinants of symptomatic versus inapparent DENV infection outcome. Future studies will show if these determinants, in particular the time interval between consecutive DENV infections, are comparable in a hyperendemic context.

Collectively, our results shed light on the factors influencing inapparent versus symptomatic DENV infection outcome. We show that while sex and infection number did not impact infection outcome, age and study year did. In the context of our long-term Pediatric Dengue Cohort Study, we were able to investigate participants with repeat DENV infections. Our results suggest that infection number (i.e., first, second, third, or post-second DENV infection) does not significantly impact inapparent versus symptomatic outcome, at least in our study. However, the time interval between a first and a second DENV infection plays a significant role in infection outcome, as a shorter interval between infections is associated with inapparent outcome. These results highlight the role of heterologous cross-protection between natural DENV infections and the importance of prospective cohort studies to study repeat DENV infections.

## Supporting Information

Checklist S1 **STROBE Checklist for cohort studies.**
(PDF)

Figure S1 **Annual sample characteristics.** (A) Distribution of the number of annual samples contributed per participant (N_participants_ = 5,541; N_samples_ = 29,090). (B) Distribution of the number of consecutive annual samples contributed per participant (N_participants_ = 5,082; N_samples_ = 28,333). (C) Distribution of the time interval between two consecutive annual samples (N_intervals_ = 23,251).
(PDF)

Figure S2 **Pre- and post-symptomatic DENV infection neutralizing titers as measured in annual samples.** For each symptomatic infection, the infecting serotype was predicted using the highest NT_50_ fold-change (in green). The serotype identified in acute samples using RT-PCR and/or virus isolation is indicated. Note that the longitudinal analysis of participant M immune history showed an inapparent DENV-2 infection prior to the symptomatic DENV-3.
(PDF)

Figure S3 **Comparison of DENV serotype circulation by neutralization assay and RT-PCR/virus isolation.** (A) DENV serotype causing symptomatic infections as determined by RT-PCR and/or virus isolation. Serotype information was available for 419 (93.6%) of 448 symptomatic infections. (B) DENV serotype causing inapparent infections as determined by neutralizing antibody titer. Serotype information was available for 73 (97.3%) of 75 inapparent infections.
(PDF)

Table S1 **Results of Participation Survey by Year in the Pediatric Dengue Cohort Study, Managua, Nicaragua, 2004–2011.**
(PDF)

Table S2 **Number of DENV infections in a subset of 39 participants of the cohort study as determined by neutralizing antibody titer.**
(PDF)
