# Supplementary material for: Symptomatic Versus Inapparent Outcome in Repeat Dengue Virus Infections Is Influenced by the Time Interval between Infections and Study Year
Source: PLoS Negl Trop Dis. 2013 Aug 8;7(8):e2357. doi: 10.1371/journal.pntd.0002357 (PMC3738476; doi:10.1371/journal.pntd.0002357)
Supplement: Figure S1 — Annual sample characteristics. (A) Distribution of the number of annual samples contributed per participant (Nparticipants = 5,541; Nsamples = 29,090). (B) Distribution of the number of consecutive annual samples contributed per participant (Nparticipants = 5,082; Nsamples = 28,333). (C) Distribution of the time interval between two consecutive annual samples (Nintervals = 23,251). (PDF) [file pntd.0002357.s002.pdf]

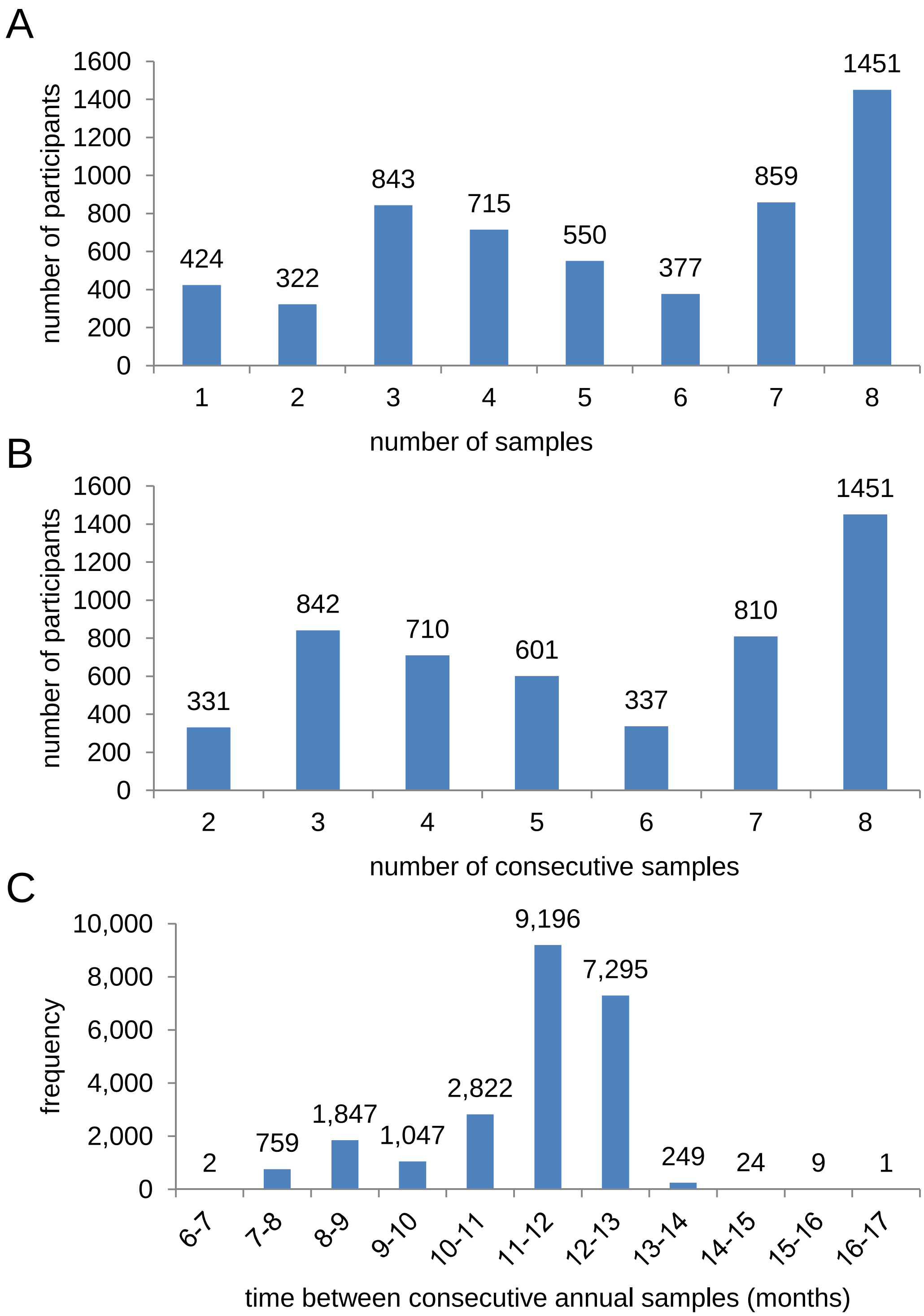

Supplementary Figure S1. Annual sample characteristics. (A) Distribution of the number of annual samples contributed per participant ($N_{participants}$=5,541; $N_{samples}$=29,090). (B) Distribution of the number of consecutive annual samples contributed per participant ($N_{participants}$=5,082; $N_{samples}$=28,333). (C) Distribution of the time interval between two consecutive annual samples ($N_{intervals}$=23,251).
