# Supplementary material for: Symptomatic Versus Inapparent Outcome in Repeat Dengue Virus Infections Is Influenced by the Time Interval between Infections and Study Year
Source: PLoS Negl Trop Dis. 2013 Aug 8;7(8):e2357. doi: 10.1371/journal.pntd.0002357 (PMC3738476; doi:10.1371/journal.pntd.0002357)
Supplement: Figure S2 — Pre- and post-symptomatic DENV infection neutralizing titers as measured in annual samples. For each symptomatic infection, the infecting serotype was predicted using the highest NT50 fold-change (in green). The serotype identified in acute samples using RT-PCR and/or virus isolation is indicated. Note that the longitudinal analysis of participant M immune history showed an inapparent DENV-2 infection prior to the symptomatic DENV-3. (PDF) [file pntd.0002357.s003.pdf]

Participant A

NT$_{50}$	Pre-infection	Post-infection	Fold change
DENV-1	46	343	7.5
DENV-2	471	2529	5.4
DENV-3	13	3442	264.8
DENV-4	29	1035	35.7
RT-PCR/virus isolation		DENV-3	

Participant C

NT$_{50}$	Pre-infection	Post-infection	Fold change
DENV-1	<10	24	4.8
DENV-2	<10	95	19.0
DENV-3	<10	11	2.2
DENV-4	<10	14	2.8
RT-PCR/virus isolation		DENV-2	

Participant D

NT$_{50}$	Pre-infection	Post-infection	Fold change
DENV-1	113	5874	52.0
DENV-2	27	238	8.8
DENV-3	<10	1382	276.4
DENV-4	27	459	17.0
RT-PCR/virus isolation		DENV-3	

Participant E

NT$_{50}$	Pre-infection	Post-infection	Fold change
DENV-1	<10	20	4.0
DENV-2	<10	54	10.8
DENV-3	24	1012	42.2
DENV-4	<10	191	38.2
RT-PCR/virus isolation		DENV-3	

Participant F

NT$_{50}$	Pre-infection	Post-infection	Fold change
DENV-1	797	4154	5.2
DENV-2	<10	1597	319.4
DENV-3	2986	8005	2.7
DENV-4	250	2183	8.7
RT-PCR/virus isolation		DENV-2	

Participant G

NT$_{50}$	Pre-infection	Post-infection	Fold change
DENV-1	47	373	7.9
DENV-2	243	475	2.0
DENV-3	36	849	23.6
DENV-4	31	551	17.8
RT-PCR/virus isolation		DENV-3	

Participant H

NT$_{50}$	Pre-infection	Post-infection	Fold change
DENV-1	49	105	2.1
DENV-2	1062	2143	2.0
DENV-3	29	2456	84.7
DENV-4	88	467	5.3
RT-PCR/virus isolation		DENV-3	

Participant I

NT$_{50}$	Pre-infection	Post-infection	Fold change
DENV-1	6192	9203	1.5
DENV-2	<10	270	54.0
DENV-3	41	3922	95.7
DENV-4	<10	129	25.8
RT-PCR/virus isolation		DENV-3	

Participant J

NT$_{50}$	Pre-infection	Post-infection	Fold change
DENV-1	47	318	6.8
DENV-2	469	2131	4.5
DENV-3	<10	1258	251.6
DENV-4	<10	552	110.4
RT-PCR/virus isolation		DENV-3	

Participant K

NT$_{50}$	Pre-infection	Post-infection	Fold change
DENV-1	52	194	3.7
DENV-2	314	353	1.1
DENV-3	42	373	8.9
DENV-4	31	105	3.4
RT-PCR/virus isolation		DENV-3	

Participant L

NT$_{50}$	Pre-infection	Post-infection	Fold change
DENV-1	2525	6333	2.5
DENV-2	<10	622	124.4
DENV-3	98	1868	19.1
DENV-4	<10	353	70.6
RT-PCR/virus isolation		DENV-2	

Participant M

NT$_{50}$	Pre-infection	Post-infection	Fold change
DENV-1	29	220	7.6
DENV-2	29	421	14.5
DENV-3	29	281	9.7
DENV-4	40	20	0.5
RT-PCR/virus isolation		DENV-3	

Participant N

NT$_{50}$	Pre-infection	Post-infection	Fold change
DENV-1	57	150	2.6
DENV-2	262	176	0.7
DENV-3	73	289	4.0
DENV-4	31	63	2.0
RT-PCR/virus isolation		DENV-3	

Participant O

NT$_{50}$	Pre-infection	Post-infection	Fold change
DENV-1	324	8830	27.3
DENV-2	62	3264	52.6
DENV-3	54	19499	361.1
DENV-4	181	3840	21.2
RT-PCR/virus isolation		DENV-3	

Participant P

NT$_{50}$	Pre-infection	Post-infection	Fold change
DENV-1	18	91	5.1
DENV-2	144	210	1.5
DENV-3	23	168	7.3
DENV-4	84	221	2.6
RT-PCR/virus isolation		DENV-3	

Participant Q

NT$_{50}$	Pre-infection	Post-infection	Fold change
DENV-1	<10	29	5.8
DENV-2	<10	552	110.4
DENV-3	<10	12	2.4
DENV-4	<10	20	4.0
RT-PCR/virus isolation		DENV-2	

Participant R

NT$_{50}$	Pre-infection	Post-infection	Fold change
DENV-1	29	83	2.9
DENV-2	34	428	12.6
DENV-3	25	35	1.4
DENV-4	<10	31	6.2
RT-PCR/virus isolation		DENV-2	

Participant S

NT$_{50}$	Pre-infection	Post-infection	Fold change
DENV-1	<10	18	3.6
DENV-2	<10	982	196.4
DENV-3	<10	63	12.6
DENV-4	97	448	4.6
RT-PCR/virus isolation		DENV-2	

Participant T

NT$_{50}$	Pre-infection	Post-infection	Fold change
DENV-1	22	390	17.7
DENV-2	102	28	0.3
DENV-3	24	31	1.3
DENV-4	45	61	1.4

RT-PCR/virus isolation: DENV-1

Participant U

NT$_{50}$	Pre-infection	Post-infection	Fold change
DENV-1	45	61	1.4
DENV-2	20	55	2.8
DENV-3	17	493	29.0
DENV-4	23	35	1.5

RT-PCR/virus isolation: DENV-3

Participant V

NT$_{50}$	Pre-infection	Post-infection	Fold change
DENV-1	21	195	9.3
DENV-2	103	256	2.5
DENV-3	26	113	4.3
DENV-4	14	37	2.6

RT-PCR/virus isolation: DENV-1

Participant W

NT$_{50}$	Pre-infection	Post-infection	Fold change
DENV-1	<10	33	6.6
DENV-2	<10	39	7.8
DENV-3	<10	225	45.0
DENV-4	<10	18	3.6

RT-PCR/virus isolation: DENV-3

Participant X

NT$_{50}$	Pre-infection	Post-infection	Fold change
DENV-1	<10	<10	1.0
DENV-2	<10	429	85.8
DENV-3	<10	<10	1.0
DENV-4	<10	<10	1.0

RT-PCR/virus isolation: DENV-2

Participant Y

NT$_{50}$	Pre-infection	Post-infection	Fold change
DENV-1	<10	<10	1.0
DENV-2	<10	2997	599.4
DENV-3	<10	44	8.8
DENV-4	<10	<10	1.0

RT-PCR/virus isolation: DENV-2

Participant Z

NT$_{50}$	Pre-infection	Post-infection	Fold change
DENV-1	<10	<10	1.0
DENV-2	102	191	1.9
DENV-3	<10	364	72.8
DENV-4	13	36	2.8

RT-PCR/virus isolation: DENV-3

Participant AA

NT$_{50}$	Pre-infection	Post-infection	Fold change
DENV-1	<10	33	6.6
DENV-2	47	745	15.9
DENV-3	<10	478	95.6
DENV-4	<10	58	11.6

RT-PCR/virus isolation: DENV-3

Participant BB

NT$_{50}$	Pre-infection	Post-infection	Fold change
DENV-1	<10	<10	1.0
DENV-2	149	192	1.3
DENV-3	<10	54	10.8
DENV-4	<10	21	4.2

RT-PCR/virus isolation: DENV-3

Supplementary Figure S2. Pre- and post-symptomatic DENV infection neutralizing titers as measured in annual samples. For each symptomatic infection, the infecting serotype was predicted using the highest NT$_{50}$ fold-change (in green). The serotype identified in acute samples using RT-PCR and/or virus isolation is indicated. Note that the longitudinal analysis of participant M immune history showed an inapparent DENV-2 infection prior to the symptomatic DENV-3.
