# Supplementary material for: Symptomatic Versus Inapparent Outcome in Repeat Dengue Virus Infections Is Influenced by the Time Interval between Infections and Study Year
Source: PLoS Negl Trop Dis. 2013 Aug 8;7(8):e2357. doi: 10.1371/journal.pntd.0002357 (PMC3738476; doi:10.1371/journal.pntd.0002357)
Supplement: Figure S3 — Comparison of DENV serotype circulation by neutralization assay and RT-PCR/virus isolation. (A) DENV serotype causing symptomatic infections as determined by RT-PCR and/or virus isolation. Serotype information was available for 419 (93.6%) of 448 symptomatic infections. (B) DENV serotype causing inapparent infections as determined by neutralizing antibody titer. Serotype information was available for 73 (97.3%) of 75 inapparent infections. (PDF) [file pntd.0002357.s004.pdf]

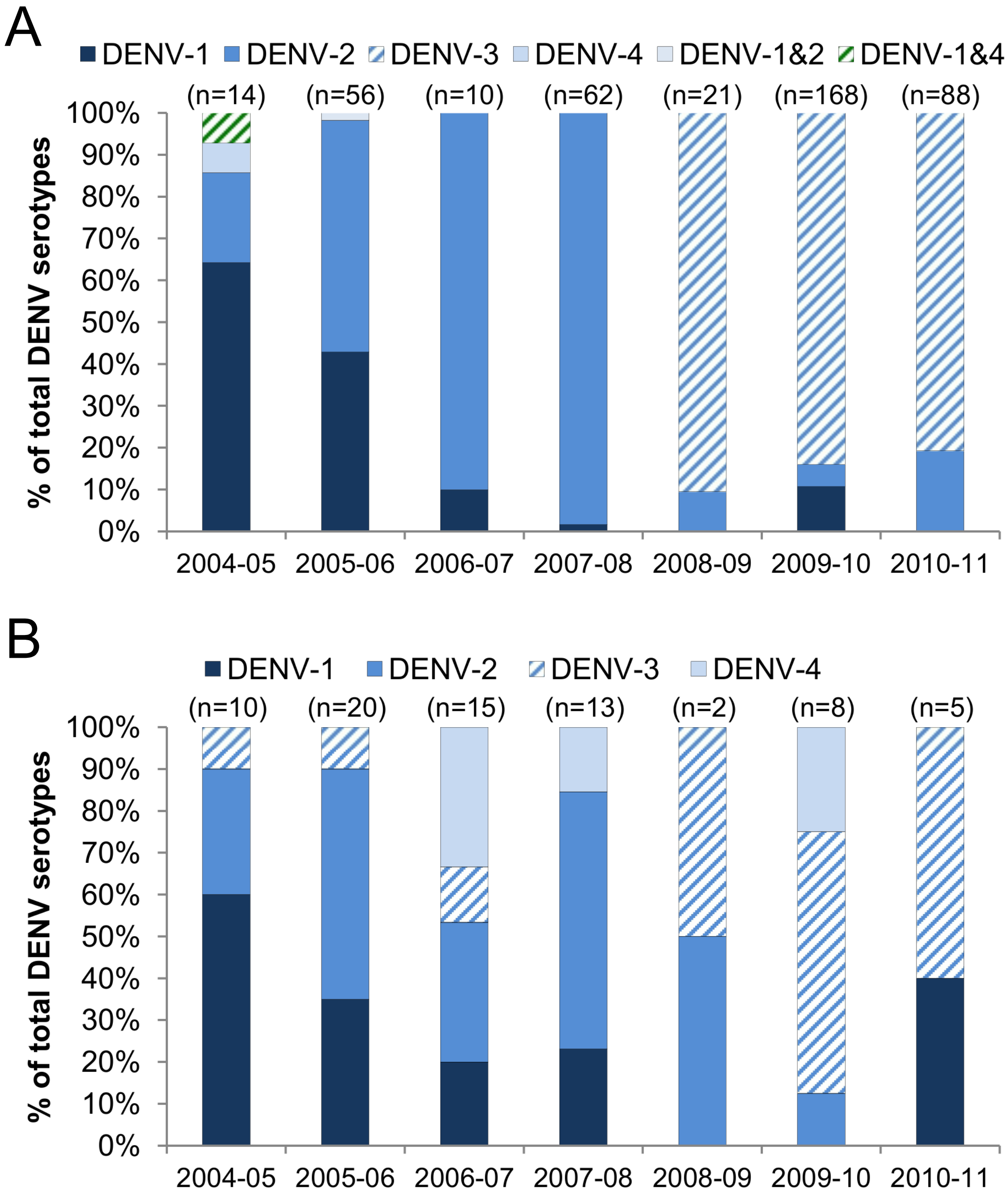

Supplementary Figure S3. Comparison of DENV serotype circulation by neutralization assay and RT-PCR/virus isolation. (A) DENV serotype causing symptomatic infections as determined by RT-PCR and/or virus isolation. Serotype information was available for 419 (93.6%) of 448 symptomatic infections. (B) DENV serotype causing inapparent infections as determined by neutralizing antibody titer. Serotype information was available for 73 (97.3%) out of 75 inapparent infections.
