# Supplementary material for: Symptomatic Versus Inapparent Outcome in Repeat Dengue Virus Infections Is Influenced by the Time Interval between Infections and Study Year
Source: PLoS Negl Trop Dis. 2013 Aug 8;7(8):e2357. doi: 10.1371/journal.pntd.0002357 (PMC3738476; doi:10.1371/journal.pntd.0002357)
Supplement: Table S1 — Results of Participation Survey by Year in the Pediatric Dengue Cohort Study, Managua, Nicaragua, 2004–2011. (PDF) [file pntd.0002357.s005.pdf]

Supplementary Table 1. Results of Participation Survey by Year in the Pediatric Dengue Cohort Study, Managua, Nicaragua, 2004–2011

	2004-05 [a] (N = 3,721)		2005-06 [a] (N = 3,695)		2006-07 [a] (N = 3,795)		2007-08 [a] (N = 3,693)		2008-09 (N = 3,953)		2009-10 (N = 3,969)		2010-11 (N = 3,866)	
	No.	%	No.	%	No.	%	No.	%	No.	%	No.	%	No.	%
Participated in survey	2,937	78.9	3,121	84.5	3,196	84.2	3,351	90.7	3,577	90.5	3,652	92.0	3,424	88.6
Had a fever and consulted other health-care provider	72	2.5	62	2.0	72	2.3	56	1.7	43	1.2	67	1.8	60	1.8
Had a fever and did not consult a medical provider	49	1.7	78	2.5	195	6.1	28	0.8	32	0.9	86	2.4	58	1.7

[a] Ref. 25
