# Supplementary material for: Symptomatic Versus Inapparent Outcome in Repeat Dengue Virus Infections Is Influenced by the Time Interval between Infections and Study Year
Source: PLoS Negl Trop Dis. 2013 Aug 8;7(8):e2357. doi: 10.1371/journal.pntd.0002357 (PMC3738476; doi:10.1371/journal.pntd.0002357)
Supplement: Table S2 — Number of DENV infections in a subset of 39 participants of the cohort study as determined by neutralizing antibody titer. (PDF) [file pntd.0002357.s006.pdf]

Supplementary Table S2. Number of DENV infections in a subset of 39 participants of the cohort study as determined by neutralizing antibody titer.

	All infections	First infection	Second infection	Third infection
DENV infections	90	36	36	18
Inapparent	75	35	27	13
Symptomatic	15	1	9	5
